# Supplementary material for: Reconciling Mining with the Conservation of Cave Biodiversity: A Quantitative Baseline to Help Establish Conservation Priorities
Source: PLoS One. 2016 Dec 20;11(12):e0168348. doi: 10.1371/journal.pone.0168348 (PMC5173368; doi:10.1371/journal.pone.0168348)
Supplement: S1 Dataset — (ZIP) [file pone.0168348.s002.zip › Taxa/Serra Norte/SN_2013/PES/Lista PESL-02.pdf]

CAVIDADE PESL-0002							
**Classe**	**Ordem**	**Fam/Outros**	**Gên/Outros**	**Espécie**	**S-U**	**U-S**	Zon.
Arachnida	Araneae	Pholcidae	*Mesabolivar*	aff. *cambridgei*		X	E
Arachnida	Araneae	Pholcidae	*Mesabolivar*	*eberhard*	X	X	E
Arachnida	Araneae	Salticidae		jovem		X	E
Arachnida	Araneae	Theridiosomatidae	*Plato*	sp.1	X		E
Arachnida	Pseudoscorpiones	Chernetidae	*Spelaeochernes*	sp.1	X		E
Arachnida	Pseudoscorpiones	Chthoniidae	*Pseudochthonius*	sp.1		X	E
Insecta	Blattodea	Blattidae		jovem		1	E
Insecta	Blattodea	Polyphagidae		jovem	1		E
Insecta	Diptera	Ceratopogonidae		jovem	X		E
Insecta	Diptera	Ephydridae		sp.	X		E
Insecta	Diptera	Psychodidae	*Psychodopygus*	*lloydi*		X	E
Insecta	Diptera	Psychodidae	*Sciopemyia*	*sordellii*		X	E
Insecta	Diptera	Psychodidae	*Trichophoromyia*	*ubiquitalis*		X	E
Insecta	Diptera	Tipulidae	Tipulinae	sp.	X		E
Insecta	Heteroptera	Reduviidae	Reduviinae	jovem	3	14	E
Insecta	Hymenoptera	Formicidae	*Camponotus*	sp.1	X	X	E
Insecta	Hymenoptera	Formicidae	*Crematogaster*	sp.2	X		E
Insecta	Isoptera	Termitidae	*Nasutitermes*	sp.	X	X	E
Insecta	Lepidoptera	Noctuoidea		sp.12	2	2	E
Insecta	Neuroptera	Mantispidae	*Plega*	sp.	1		E
Insecta	Neuroptera	Myrmeleontidae		jovem	3	2	E
Malacostraca	Isopoda	Armadillidae		sp.1		X	E
Mammalia	Chiroptera	Emballonuridae	*Peropteryx*	sp.	3	1	E
